# 970 Discussion of Management of Burn Unit Shortages; Compendium of Solutions from (ABA) Burn Surgeon Forum

**DOI:** 10.1093/jbcr/iraf019.501

**Published:** 2025-04-01

**Authors:** Roselle Crombie, Alexandra Lacey, Kathleen Romanowski, Anju Saraswat

**Affiliations:** Connecticut Burn Center, Bridgeport Hospital / Yale-New Haven Health; Regions Hospital Burn Center; University of California Davis; Atrium Health Wake Forest Baptist

## Abstract

**Introduction:**

The impact of COVID-19 continues to have profound effects, even 4 years after the pandemic. Despite many efforts to return to pre-pandemic norms, healthcare is still facing ongoing shortages of essential drugs, dressings, and instruments needed in burn care. This new reality is driven by persistent supply chain disruptions and significant revenue losses experienced during COVID, which have resulted in the selective unavailability of key treatment modalities.

**Methods:**

The annual Burn Surgeon Forum (BSF) is now in its third year. To better understand the scope of regional and national shortages, various burn units across the country were asked to provide an updated list of pharmacy shortages immediately prior to the conference. During the BSF, additional input from participants was included in a robust discussion that took place over two days, with two separate breakout sessions. The cohort of participants represented a diverse group from multiple geographic regions and included surgeons at various career stages—junior, mid-career, and senior.

**Results:**

A variety of ideas regarding drug shortages and possible alternatives were shared. An abridged list of shortages and proposed solutions is presented in Table 1. In addition to discussing specific shortages and their solutions, participants explored broader strategies to mitigate these challenges. Ideas included fostering partnerships between burn units, addressing metacognitive gaps in problem-solving, and improving direct communication between the American Burn Association (ABA) and its members to ensure rapid dissemination of critical information.

Shortage Solution

Mafenide Suspend Cream / Alternative topical treatments

Oxandrolone Testosterone / Growth hormone / Propranolol

Mineral Oil Saline / Hibiclens / Surgilube

**Conclusions:**

Four years after the onset of the COVID-19 pandemic, the healthcare system still faces significant shortages in drugs and equipment, especially within burn care. By randomly grouping burn surgeons from different regions and stages of training, the Burn Surgeon Forum facilitated a robust and productive discussion that yielded several practical solutions. We aim to share the outcomes of these discussions at a larger venue, such as our upcoming annual meeting, to further engage with the broader medical community.

**Applicability of Research to Practice:**

Drug and equipment shortages are ubiquitous. Different burn centers have established unique solutions to these issues. By sharing these solutions, all of our patients can benefit.

**Funding for the Study:**

N/A

